# Influence of Procedure Type on Time to Urethoplasty: A Retrospective Comparison of Urethral Dilatation and Direct Visual Internal Urethrotomy for Anterior Urethral Strictures

**DOI:** 10.7759/cureus.117619

**Published:** 2026-10-07

**Authors:** Abdalhakim S Nimate, Sofian F AlBdour, Muhammad Rawashdeh, Mohammad Nusir, Zaid Alqudah, Talal A Musa

**Affiliations:** 1 Urology, King Hussein Medical City, Amman, JOR; 2 Urology, King Hussein Medical Center, Amman, JOR

**Keywords:** direct visual internal urethrotomy, urethral dilatation, urethral stricture, urethral trauma, urethroplasty

## Abstract

Background: Anterior urethral strictures are managed in various ways, and endoscopic treatment remains the most common, despite limited durability. Compared to definitive reconstruction, the evidence supporting the role of endoscopic techniques is vague regarding their influence on disease progression.

Objective: This study aims to compare urethral dilatation and direct visual internal urethrotomy (DVIU) in terms of time to urethroplasty.

Methods: This retrospective cohort analysis was conducted at Prince Hussein Urology and Organ Transplant Centre, King Hussein Medical Centre, Amman, Jordan, between 2020 and 2025. Adult patients with anterior urethral strictures treated with urethral dilatation (Group I) or DVIU (Group II) were included. Prior urethroplasty and incomplete follow-up were exclusion criteria. The primary outcome measure was time to urethroplasty, defined as the number of months between the index endoscopic procedure at our institution to treat urethral stricture and its first substitution urethroplasty (definitive urethral reconstruction in which a tissue graft or flap was used to augment or replace the diseased segment of the urethra). Patients were followed for a minimum of 12 months and censored at last follow-up if urethroplasty had not been performed. Survival analysis was performed using Kaplan-Meier methods with log-rank testing, and Cox proportional hazards regression was used to identify independent predictors.

Results: A total of 193 patients were included, with 73 (37.8%) in Group I and 120 (62.2%) in Group II. Baseline characteristics were similar, but Group I had a higher proportion of patients with multiple prior endoscopic procedures (p=0.041). Idiopathic strictures were the most common etiology (47.2%). Urethroplasty was required in 31.5% of Group I and 25.8% of Group II patients (p=0.389). There was no difference in urethroplasty-free survival (log-rank p=0.403). In multivariable analysis, two or more prior endoscopic procedures were independently associated with shorter time to urethroplasty (adjusted HR 4.286, p<0.001).

Conclusions: The frequency of previous endoscopic treatments, rather than the choice of initial technique, was strongly associated with progression to urethroplasty. Urethral dilatation and DVIU yielded comparable outcomes.

## Introduction

Strictures of the anterior urethra remain a common cause of lower urinary tract obstruction and continue to pose a serious clinical burden. The estimated prevalence is approximately 200-600 cases per 100,000 men, with incidence increasing with age and comorbidity [1]. Patients most commonly present with progressive voiding symptoms and recurrent urinary tract infections and are prone to repeated interventions over a prolonged disease course.

The etiologies are varied and have changed over time․ The most common etiologies of urethral stricture were idiopathic (34%) and iatrogenic (32%), followed by inflammatory (20%) and traumatic (14%) etiologies. Common iatrogenic etiologies included long-term catheterization and prior transurethral procedures․ The bulbar urethra was the most commonly involved area (52%) [2]. 

Treatment planning requires accurate stricture characterization. Evaluation typically combines symptom assessment alongside uroflowmetry and anatomical definition using retrograde urethrography. Traditional approaches have expanded beyond imaging to include endoscopic assessment and adjunctive diagnostic techniques, allowing more precise delineation of stricture length, location, and severity [3]. Collectively, these investigations are necessary for selecting appropriate management strategies.

Most approaches involve endoscopic evaluation initially. Given the simplicity and accessibility of urethral dilatation and direct visual internal urethrotomy (DVIU), they are the most widely practiced. Survey data indicate that urethral dilatation is used by approximately 92.8% of urologists, while 85.6% use cold-knife urethrotomy. In contrast, other minimally invasive modalities, including endourethral stents (23.4%), laser urethrotomy (19%), and periurethral steroid injection (7.9%), are used far less often [4].

Although their use is widespread, the durability of repeated endoscopic intervention is limited. Clinical evidence demonstrates that repeat urethrotomy or dilatation provides little sustained benefit, with diminishing effectiveness over time, and is neither clinically effective nor cost-effective. Such strategies may also delay definitive management without improving long-term outcomes [5].

Recurrence after endoscopic treatment remains a central limitation. Reported recurrence rates range from 30% to 60% following an initial urethrotomy and increase to 50% to 100% after subsequent procedures. Repeated transurethral manipulation has been associated with increasing stricture complexity and prolonged disease duration, likely reflecting cumulative tissue injury and progressive spongiofibrosis. Furthermore, prior endoscopic treatment and intermittent self-dilatation have been shown to adversely influence outcomes following urethroplasty [6].

Given these observations, the role of endoscopic management within the treatment pathway remains incompletely defined. In particular, it is unclear whether the initial choice between urethral dilatation and DVIU influences progression to definitive reconstruction. While recurrence following endoscopic treatment is well established, its impact on the timing of urethroplasty has not been adequately addressed. This study, therefore, evaluates the effect of these two commonly used techniques on time to urethroplasty and identifies factors associated with progression to definitive surgical management.

## Materials and methods

This retrospective cohort study was conducted at Prince Hussein Urology and Organ Transplant Centre, King Hussein Medical City, Amman, Jordan, between January 2020 and December 2025, following approval from the institutional review board. This study was approved by the Ethical Committee, Jordanian Royal Medical Services, under approval number 49/10/2026. All data were handled in accordance with applicable ethical standards and institutional data protection regulations.

Adult male patients (≥18 years) with symptomatic anterior urethral stricture who underwent endoscopic management with either urethral dilatation or DVIU were eligible for inclusion. Diagnosis was established using retrograde urethrogram and cystoscopy. The index procedure was defined as the first endoscopic intervention performed at our institution during the study period, following an appropriate period of urethral rest when indicated, and represented time zero for the time-to-event analysis. Previous endoscopic procedures performed before the index intervention were recorded separately and incorporated into the analysis as a treatment history variable. A minimum follow-up duration of 12 months was required for meaningful assessment of outcomes.

A total of 193 patients met the inclusion criteria and were included in the analysis, of whom 73 underwent urethral dilatation and 120 underwent DVIU.

Patients were excluded if they had posterior urethral strictures, including those related to pelvic fracture urethral injury, prior urethroplasty, urethral malignancy, or incomplete clinical or follow-up data. Patients managed with suprapubic catheterization without subsequent definitive endoscopic treatment were also excluded.

Data were retrieved from the national electronic medical record system (Hakeem, Electronic Health Solutions, Amman, Jordan), with verification against operative records and radiological archives where necessary. Data extraction was performed using a standardized collection framework to ensure consistency. Patients for whom definitive reconstruction was selected as the initial management were excluded. Cases with missing key variables, particularly those affecting the primary outcome or follow-up duration, were excluded. For variables with limited missing data not influencing the primary endpoint, analyses were conducted on available cases without imputation.

Preoperative assessment included clinical history, physical examination, and imaging with retrograde urethrogram. Urinary tract infection was excluded prior to intervention, and when present, treated before proceeding. Patients presenting with acute urinary retention were managed initially with suprapubic catheterization, followed by a period of urethral rest prior to definitive endoscopic treatment.

For urethral dilatation, the stricture was traversed using a hydrophilic guide-wire under direct visualization. Progressively larger dilators were used in sequence based on the specific nature of the stricture and surgeon preference․ In DVIU‚ the stricture was incised under direct visualization‚ usually at the 12 o'clock position‚ to achieve an acceptable degree of opening․ A catheter was placed in the urethra after both types of procedures for an undetermined period of time based on the surgeon's assessment during surgery․ Other procedures performed at the same time as the initial procedure are noted if mentioned in the operative report.

The study was designed to compare two endoscopic treatment techniques and was not a comparison of endoscopic treatment with primary urethroplasty. Selection of the endoscopic technique and subsequent consideration of urethroplasty were based on routine clinical decision-making by the treating urologist and were not assigned by study protocol. Taking into account stricture characteristics, previous treatment, clinical response, and patient preference when documented. Urethroplasty was recommended for patients who required more definitive repair during follow-up, especially those who experienced ongoing or recurrent obstruction despite endoscopic treatment.

Patients followed up at three, six, and 12 months after their initial procedure and then followed up periodically until either urethroplasty or the date of final recorded follow-up. For those who did not have urethroplasty, the date of the final recorded follow-up was considered a censored observation for survival analysis. Follow-up evaluation included assessment of lower urinary tract symptoms, with uroflowmetry performed when available. Further investigations, including imaging or cystoscopy, were undertaken when recurrence was suspected based on clinical or functional decline.

Prior to collection, variables were defined before analysis and selected based on clinical relevance alongside previous evidence. These included age, diabetes mellitus, hypertension, and current smoking status. Stricture characteristics implied anatomical location, stricture length measured on imaging, and cause of stricture categorized as idiopathic, iatrogenic, traumatic, or inflammatory. Treatment-related variables included the type of endoscopic procedure and number of previous endoscopic interventions, categorized as fewer than two versus two or more, as treatment-related variables.

The primary outcome investigated was time to urethroplasty, defined as the interval in months between the index endoscopic procedure and subsequent substitution urethroplasty. In this study, substitution urethroplasty referred to definitive urethral reconstruction in which a tissue graft or flap was used to augment or replace the diseased urethral segment. Patients who did not undergo urethroplasty were censored at the time of last clinical follow-up.

IBM SPSS Statistics for Windows, version 27 (IBM Corp., Armonk, NY, USA), was used to perform statistical analysis. Continuous variables were assessed primarily for distribution and presented as mean ± standard deviation or median with interquartile range as appropriate. Frequencies and percentages were used to express categorical variables and compared using the chi-square test or Fisher’s exact test, while continuous variables were compared using independent t-tests or Mann-Whitney U tests as appropriate.

Urethral stricture length was studied as a measured continuous variable and as a categorical variable using a 3-cm cutoff that was clinically meaningful, according to urethroplasty studies. Time to urethroplasty was analyzed using Kaplan-Meier methods, and time to urethroplasty was compared between treatment groups using the log-rank test. Time to urethroplasty was censored for some patients, so Kaplan-Meier estimates and the log-rank test were used to compare time to urethroplasty instead of observed times to urethroplasty or other standard comparisons. Cox proportional hazards regression was utilized to evaluate factors associated with time to urethroplasty. For regression analysis, stricture etiology was treated as a categorical variable, with idiopathic stricture used as the reference category; HRs were estimated separately for iatrogenic, traumatic, and inflammatory strictures. Variables with a p-value <0.20 in univariable analysis were considered for inclusion in the multivariable model. Procedure type, the primary exposure of interest, and prior endoscopic treatment burden were retained in the multivariable model irrespective of their univariable p-values because of their potential confounding effect on the primary comparison. The proportional hazards assumption was assessed using Schoenfeld residuals and inspection of log-minus-log plots, and multicollinearity was evaluated using variance inflation factors. Adjusted HRs with 95% CIs were reported, and statistical significance was defined as a two-sided p-value <0.05.

A formal, planned sample size calculation was not conducted for this study because it was a retrospective study. All study-eligible patients who met predefined criteria during the study period were included․ A post hoc power calculation was conducted to assess the power to find a difference in the rate of urethroplasty procedures between the two treatment groups using a two-sided α of 0․05 and the proportions of patients in each group at the end of the study․ The difference between the event rates observed in this study was small, and the power calculation indicated low power to detect a difference. Findings from this study were interpreted while taking a type II error into account

All variables were defined prior to analysis to maintain methodological consistency and reduce the risk of bias.

## Results

A total of 193 patients were included in the study. Group I (urethral dilatation) encompassed 73 patients (37.8%), while Group II (DVIU) included 120 patients (62.2%). Table 1 summarizes baseline characteristics and stricture etiology. The two groups were comparable in terms of age, comorbidities, and stricture characteristics. Group I had a higher proportion of patients that have undergone two or more prior endoscopic procedures, representing the only statistically significant baseline difference. Idiopathic strictures were the most common (47.2%), followed by iatrogenic (29.0%) and traumatic causes (20.2%), while inflammatory strictures were uncommon (3.6%). The anatomical locations of urethral strictures were similar in the two treatment groups․ The most common types of strictures were bulbar and penile, followed by penobulbar and meatal or fossa navicularis strictures. Bulbar strictures were identified in 41 (56․2%) of patients in the dilation group and 68 (56․7%) of patients in the DVIU group․ Penile strictures were seen in 13 (17․8%) and 21 (17․5%) of patients, respectively. Rates of penobulbar and meatal or fossa navicularis strictures were similar in both groups, and there was no significant difference in the rate of strictures at other anatomical sites (p=1․000).

**Table 1:** Baseline characteristics of patients according to procedure type DVIU: direct visual internal urethrotomy

Variable	Dilatation (n=73)	DVIU (n=120)	Test Used	p-value
Age (years, mean ± SD)	48.2 ± 15.1	48.8 ± 14.7	Independent t-test	0.812
Diabetes mellitus (%)	19 (26.0%)	29 (24.2%)	Chi-square	0.768
Hypertension (%)	25 (34.2%)	43 (35.8%)	Chi-square	0.821
Smoking (%)	24 (32.9%)	38 (31.7%)	Chi-square	0.859
Stricture length (cm, mean ± SD)	3.2 ± 1.5	3.0 ± 1.6	Mann–Whitney U	0.284
Stricture length >3 cm (%)	37 (50.7%)	56 (46.7%)	Chi-square	0.598
≥2 prior procedures (%)	38 (52.1%)	48 (40.0%)	Chi-square	0.041
Cause of stricture			Chi-square	0.402
Idiopathic	36 (49.3%)	55 (45.8%)		
Iatrogenic	25 (34.2%)	31 (25.8%)		
Traumatic	10 (13.7%)	29 (24.2%)		
Inflammatory	2 (2.7%)	5 (4.2%)		

Univariable Cox regression analysis is presented in Table 2. Increased hazard of progression to urethroplasty coincides with the presence of two or more prior procedures, regardless of type (HR 4.319, p<0.001), whereas diabetes mellitus showed a non-significant trend (HR 1.676, p=0.067). On the contrary, no associations were observed for age, hypertension, smoking status, stricture length, procedure type, or cause of stricture.

**Table 2:** Univariable Cox proportional hazards analysis for time to urethroplasty Idiopathic stricture was used as the reference category.

Variable	HR	95% CI	p-value
Age (per year)	1.012	0.997 – 1.028	0.122
Diabetes mellitus	1.676	0.965 – 2.912	0.067
Hypertension	1.159	0.667 – 2.014	0.600
Smoking	1.000	0.659 – 1.517	0.999
Stricture length (per cm)	1.040	0.877 – 1.234	0.650
Stricture length >3 cm	1.139	0.676 – 1.919	0.623
≥2 prior procedures	4.319	2.225 – 8.382	<0.001
Procedure type (direct visual internal urethrotomy vs. dilatation)	0.797	0.465 – 1.367	0.409
Cause of stricture: iatrogenic vs. idiopathic; traumatic vs. idiopathic; inflammatory vs. idiopathic	1.041	0.773-1.403	0.789

After adjustment for prior endoscopic treatment burden, procedure type was not significantly associated with time to urethroplasty (adjusted HR 0.941, 95% CI 0.547-1.1619; p=0.826).In contrast, patients with two or more prior endoscopic procedures had a significantly shorter time to urethroplasty (adjusted HR 4.286, 95% CI 2.201-8.341; p<0.001) (Table 3).

**Table 3:** Multivariable cox proportional hazards analysis for time to urethroplasty Procedure type was retained in the multivariable model as the primary exposure. Prior endoscopic treatment burden was included to account for the baseline imbalance between treatment groups.

Variable	Adjusted HR	95% CI	p-value
Age	1.010	0.994-1.027	0.205
Diabetes	1.589	0.902-2.801	0.108
≥2 prior procedures	4.286	2.201-8.349	<0.001
Procedure type (direct visual internal urethrotomy vs. dilatation)	0.941	0.547-1.619	0.826

Outcome measures are shown in Table 4․ Although 23 (31․5%) patients in Group I and 31 (25․8%) patients in Group II needed subsequent urethroplasty‚ this difference was not statistically significant (p=0․389)․ Kaplan-Meier analysis showed equivalent risk of subsequent urethroplasty in the two groups․ Log-rank test results were not statistically significant (p=0․403)․

**Table 4:** Outcome summary by procedure type ¹Chi-square test; ^2^Log-rank test (Kaplan–Meier analysis); DVIU: direct visual internal urethrotomy

Outcome	Dilatation (n=73)	DVIU (n=120)	p-value
Urethroplasty events, n (%)	23 (31.5%)	31 (25.8%)	0.389¹
Urethroplasty-free survival, Kaplan–Meier analysis	—	—	0.403^2^

Time-to-event analysis illustrated comparable urethroplasty-free survival between groups. Kaplan-Meier curves (Figure 1) showed overlapping survival trajectories throughout follow-up, with no statistically significant difference on log-rank testing (p=0.403).

**Figure 1:**
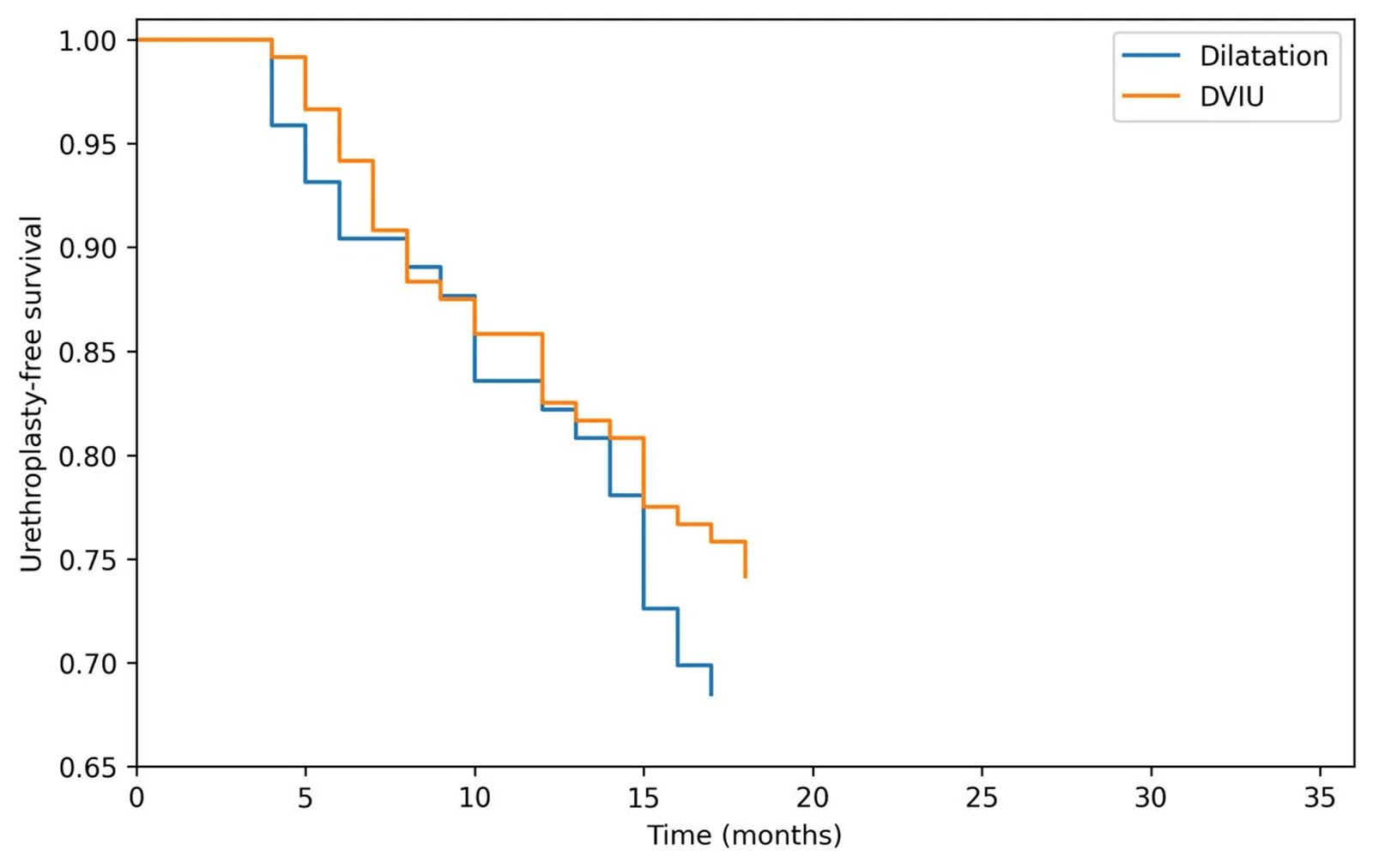
Kaplan–Meier curves for urethroplasty-free survival in patients treated with urethral dilatation versus direct visual internal urethrotomy (DVIU; log-rank p = 0.403).

Overall, progression to urethroplasty was independently associated with prior endoscopic treatment burden, while procedure type and stricture etiology were not independent predictors of time to urethroplasty.

## Discussion

This study examined whether the index endoscopic procedure performed at our institution, urethral dilatation (Group I) or DVIU (Group II), influenced progression to urethroplasty. Because a substantial proportion of patients had undergone previous endoscopic procedures, treatment history was considered separately from the index procedure in the analysis. This analysis should not be interpreted as an evaluation of whether endoscopic treatment should be preferred over primary urethroplasty. Our study demonstrates no difference in progression to definitive reconstruction or durability of treatment, as both techniques had comparable outcomes. Although this supports the previously published evidence on long-term comparable outcomes of both dilation and urethrotomy, it shows a tendency toward recurrence and the need for further intervention [7]. Clinically, both approaches are similarly limited. Disrupting the stricture and restoring luminal patency is the mechanism behind both dilatation and DVIU, but they leave the underlying fibrotic process unaddressed. As a result, their long-term durability is limited, and the recurrence rate is high, particularly with repeated procedures. This shared mechanism likely explains the similar clinical course shown in the current series [8]. 

An important factor affecting the comparison of the two endoscopic procedures is the difference in past treatments between patients receiving the two procedures. A higher percentage of dilatation patients than DVIU patients had received two or more previous endoscopic procedures (52% vs. 40%, p=0.041). Past endoscopic procedures were strongly correlated with subsequent urethroplasty in this study population and likely affected observed differences between groups in time to urethroplasty․ After adjusting for past procedures, the endoscopic procedure was not correlated with time to urethroplasty. These results indicate that differences in past procedures, rather than endoscopic procedures used, may explain differences in outcomes, though the study design does not allow conclusions about causation [6]. 

Metabolic factors seem to have a limited role in this study. Diabetes showed a modest association on initial analysis, yet it proved to be a poor determinant of progression. This supports the findings indicating that metabolic dysfunction could play a role in slowing healing and promoting fibrosis; however, its overall influence is not as prominent as that of recurrent mechanical intervention [9]. 

Unlike our results, Kinnaird et al. [10] found evidence of higher recurrence rates after urethroplasty in patients with certain stricture etiologies, specifically infectious and iatrogenic causes and lichen sclerosus. In our study, we did not find an association between stricture etiology and time to urethroplasty. This difference may be due to the different outcomes assessed by the two studies. Kinnaird et al. examine recurrence after urethroplasty [10]‚whereas we examine progression to urethroplasty after endoscopic therapy.

Overall, these findings support that treatment is substantially affected by treatment. Differences between dilation and DVIU were outweighed by the added strain of prior interventions in dictating long-term outcomes, parallel to broader evidence stressing the multifactorial nature of urethral stricture disease [11].

On one hand, the clinical perspective grounds selective approaches to repeated endoscopic management based on these findings. On the other hand, routine practice showed that referral for urethroplasty would be a last resort after having undergone multiple procedures despite the limited durability of these interventions. Earlier consideration of urethroplasty, notably in patients with prior interventions, may limit disease escalation and reduce overall treatment burden [12]. 

These results are compatible with those of a randomized trial by Steenkamp et al., which compared endo-urethrotomy to urethral dilation and found no significant differences between the two procedures in terms of outcomes measured [13].

This study had a defined study population and clinically relevant outcome of interest, progression to urethroplasty, in addition to recurrence. Time-to-event analyzes support the conclusions․ Study limitations include the retrospective design․ It was underpowered to detect a difference in outcomes between the two endoscopic procedures of interest․ The actual difference in urethroplasty events between the two procedures was small․ A post hoc power analysis suggested the study would have had little power to detect a difference․ Therefore, no significant difference should be interpreted as a lack of evidence of a difference, not as evidence of equivalence between the two procedures. Other limitations include potential selection bias, single-center recruitment, and lack of complete standardized information regarding the timing and specific characteristics of all endoscopic procedures performed before the index intervention. 

## Conclusions

Urethral dilatation and DVIU had similar outcomes in progression to urethroplasty. In multivariate analysis, urethral dilatation and DVIU were not significantly different in their effects on time to urethroplasty. A history of two or more prior endoscopic procedures was significantly associated with a shorter time to urethroplasty. Causation should not be assumed from these results because of the retrospective study design․
